# Supplementary material for: Molecular basis of differential 3′ splice site sensitivity to anti-tumor drugs targeting U2 snRNP
Source: Nat Commun. 2017 Dec 13;8:2100. doi: 10.1038/s41467-017-02007-z (PMC5727392; doi:10.1038/s41467-017-02007-z)
Supplement: Supplementary file 1 — Supplementary Information [file 41467_2017_2007_MOESM1_ESM.pdf]

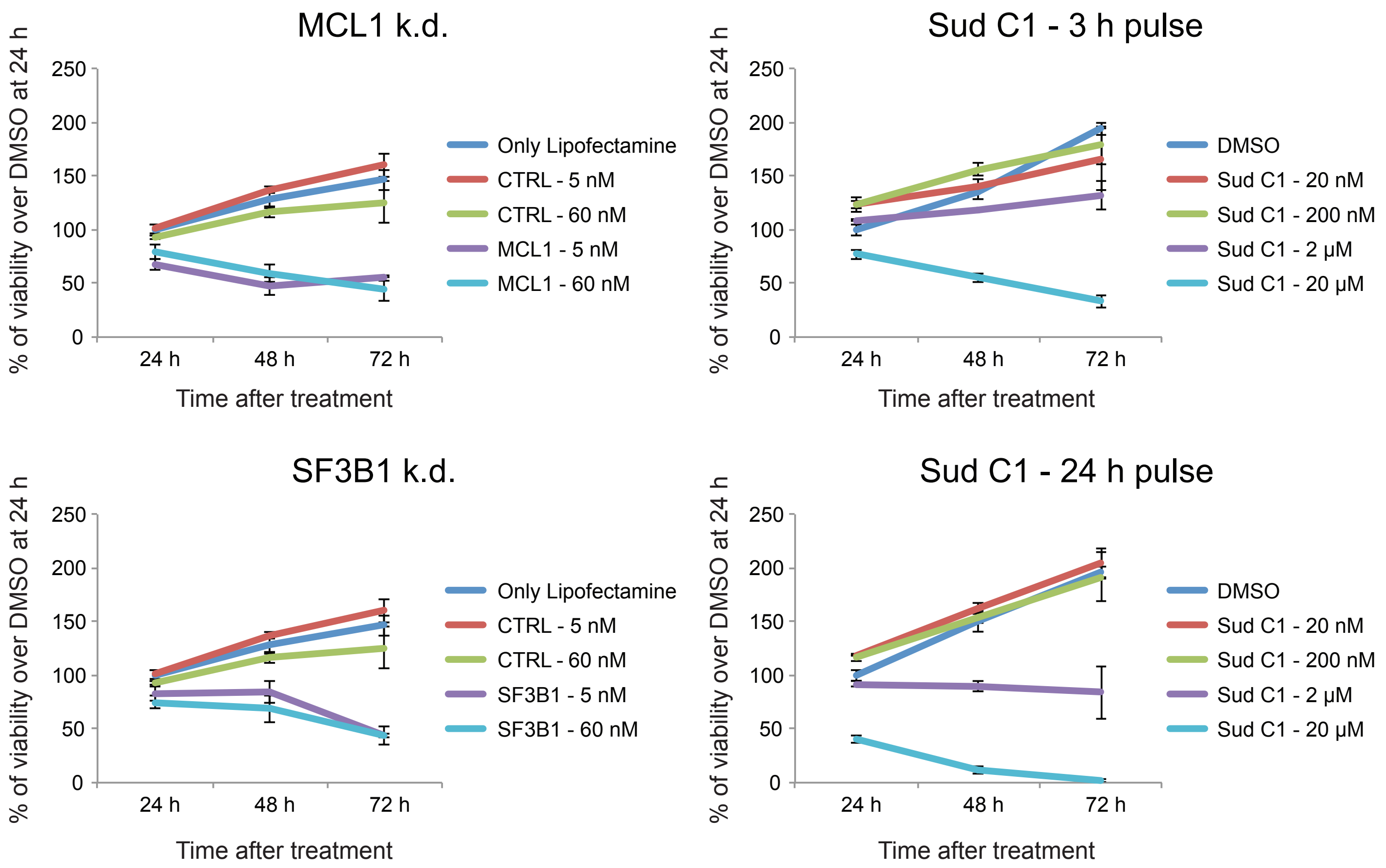

Supplementary Figure 1. MCL1 and SF3B1 levels are important to maintain cell viability. Viability of HeLa cells was measured using Resazurin assays upon MCL1 or SF3B1 knock down by RNAi, using the indicated concentrations of siRNAs (left panels) or upon treatment with the indicated concentrations of Sud C1. Measurements were taken 24, 48 and 72 hours after the different treatments. CTRL: scrambled siRNA.

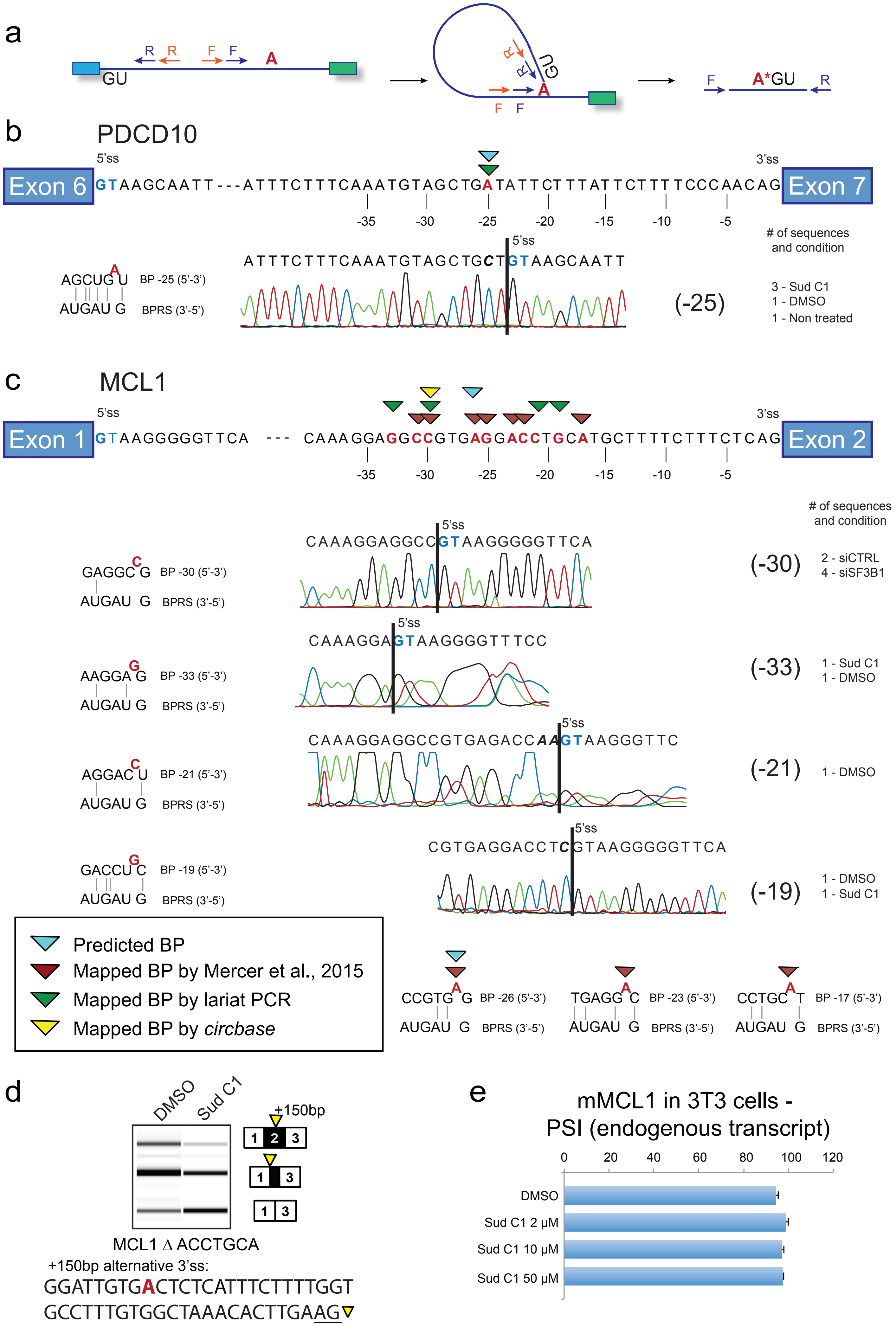
a
GU
R R F F
A
GU
A
A*GU
b
PDCD10
5'ss
3'ss
Exon 6
GTAAGCAATT---ATTTCTTTCAAATGTAGCTGATATTCTTTATTCTTTTCCCAACAG
Exon 7
-35 -30 -25 -20 -15 -10 -5
# of sequences and condition
ATTTCTTTCAAATGTAGCTGCTGTAAGCAATT
AGCUG A U BP -25 (5'-3')
AUGAU G BPRS (3'-5')
(-25)
3 - Sud C1
1 - DMSO
1 - Non treated
c
MCL1
Exon 1
GTAAGGGGGTTCA --- CAAAGGAGGCCGTGAGGACCTGCATGCTTTTCTTTCTCAG
Exon 2
CAAAGGAGGCCGTAAGGGGGTTCA
GAGGC C G BP -30 (5'-3')
AUGAU G BPRS (3'-5')
(-30)
2 - siCTRL
4 - siSF3B1
CAAAGGAGTAAGGGGTTTCC
AAGGA G G BP -33 (5'-3')
AUGAU G BPRS (3'-5')
(-33)
1 - Sud C1
1 - DMSO
CAAAGGAGGCCGTGAGACCAAGTAAGGGTTC
AGGAC C U BP -21 (5'-3')
AUGAU G BPRS (3'-5')
(-21)
1 - DMSO
CGTGAGGACCTCGTAAGGGGGTTCA
GACCU G C BP -19 (5'-3')
AUGAU G BPRS (3'-5')
(-19)
1 - DMSO
1 - Sud C1
Predicted BP
Mapped BP by Mercer et al., 2015
Mapped BP by lariat PCR
Mapped BP by *circbase*
CCGTG A G BP -26 (5'-3')
AUGAU G BPRS (3'-5')
TGAGG A C BP -23 (5'-3')
AUGAU G BPRS (3'-5')
CCTGC A T BP -17 (5'-3')
AUGAU G BPRS (3'-5')
d
DMSO
Sud C1
+150bp
1 2 3
1 3
1 3
MCL1 Δ ACCTGCA
+150bp alternative 3'ss:
GGATTGTGACTCTCATTTCTTTTGGT
GCCTTTGTGGCTAAACACTTGAAG
e
mMCL1 in 3T3 cells -
PSI (endogenous transcript)
0 20 40 60 80 100 120
DMSO
Sud C1 2 μM
Sud C1 10 μM
Sud C1 50 μM

Supplementary Figure 2. *PDCD10* intron 6 has a well-defined BP, while *MCL1* intron 1 has multiple weak BPs.

a) Scheme of the position of oligonucleotides for RT-PCR assays and expected amplification products. The branch A is often mutated in this assay (as indicated by an asterisk in the scheme). b) BP mapping of *PDCD10* intron 6 lariats using two different cell lines. One representative electropherogram of the amplification products is shown. 5' terminal GT sequences corresponding to the intron 5' end are shown in blue. Vertical bars indicate split between sequences at the 3' end of the intron and the 5' splice site; the split should be diagnostic of the position of the BP, with 1-2 nucleotide resolution[1,2]. The cell line, the numerical positions of the putative BPs (nearest adenosine to the split position), the number of sequenced products and the treatment condition are indicated on the right of each result. Schemes of base-pairing complementarity with U2 snRNA corresponding to the potential BPs are also indicated. c) BP mapping of *MCL1* intron 1 lariats, carried out in HeLa cells as in (b) Various potential BPs were detected, suggesting degenerate BP usage, consistent with a previous report[2]. The most probable computationally inferred BPs within the last 60 nt of the intron are shown[3]: PDCD10 BP scoring value svm_scr is 1.34, MCL1 BP scoring value svm_scr is -0.34, highlighting the weakness of MCL1 intron 1 BPs. At the bottom of the figure, base-pairing with U2 snRNA are shown for positions previously mapped[2] and predicted by SVM_BP[3]. A mapped BP from a circular RNAs database that also contains some lariat sequences (http://www.circbase.org/)[4] is shown for *MCL1* (hsa_circ_0002364 annotated circular RNA). d) Representative capillary electrophoresis profile of *MCL1* transcript isoforms from a minigene lacking the -23/-17 ACCTGCA sequence. Activation of an exonic cryptic 3’ss (at position +150 from the conventional one) was observed and confirmed by Sanger sequencing. e) Regulation of endogenous *MCL1* in mouse 3T3 cells treated with the indicated amounts of Sud C1.

a

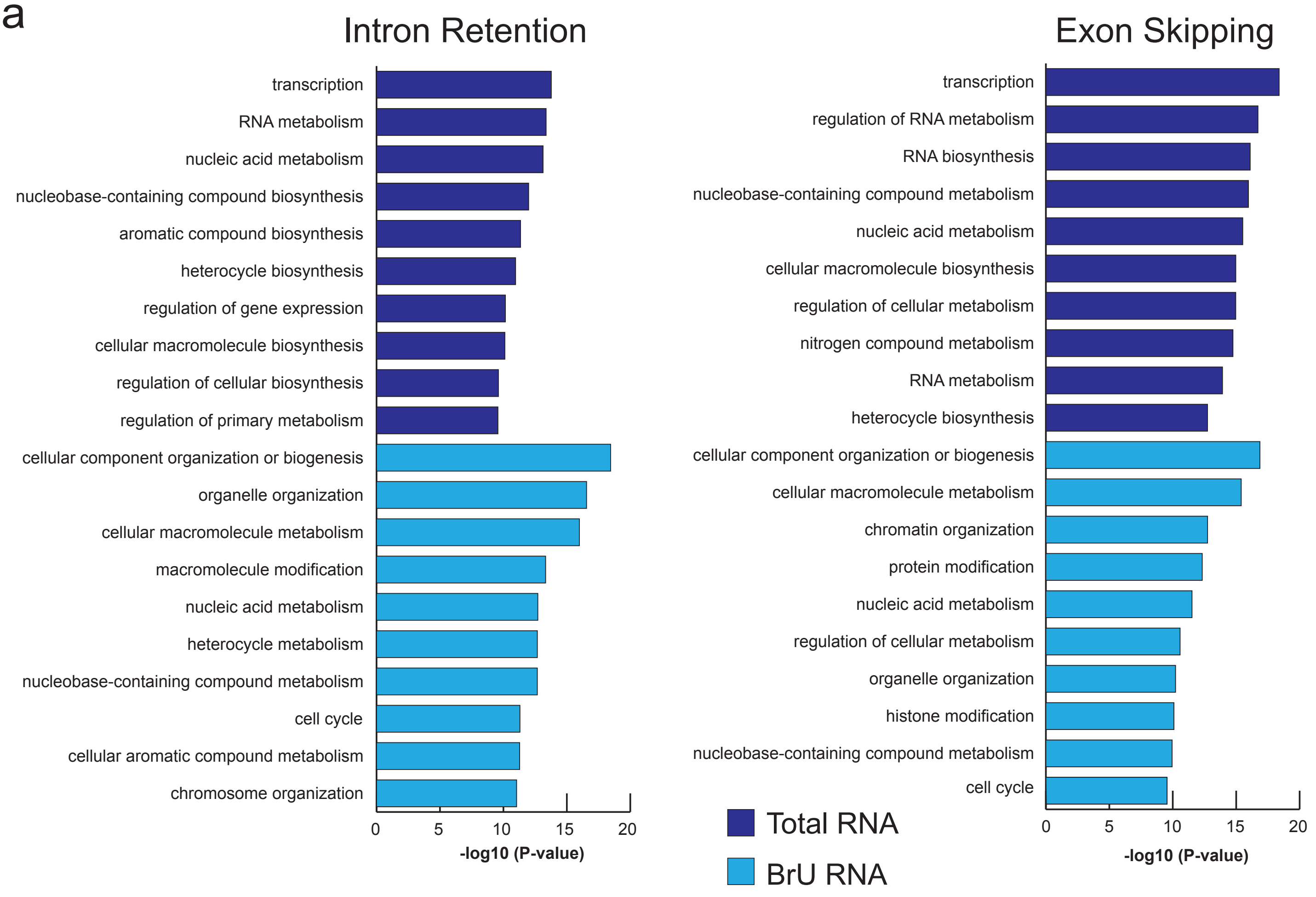

b

Spliceosome Components with at least 15% DPSI in SudC1-treated cells (either total RNA or BrU-RNA)

A complex		B and Bact complex					C and P complex		Major		
CCAR1	SF3A3	AQR	CWC25	LSM5	PRCC	SNRNP40	C9orf78	MAGOH	C16orf80	GNB2L1	PRPF4B
DDX46	SF3B2	BCAS2	CWC27	LSM6	PRPF19	SNW1	CDK10	NOSIP	CCDC130	JUP	RBM4B
DHX15	SF3B3	BUD13	DDX23	MFAP1	PRPF3	TFIP11	CXorf56	PPIG	CCDC75	KIAA1967	RUVBL1
HNRNPAB	SNRNP70	BUD31	DHX16	NAA38	PRPF31	TXNL4A	DDX41	PPIL3	CCDC94	KIN	SART3
HTATSF1	SNRPC	CCDC12	EFTUD2	NHP2L1	PRPF38A	USP39	DGCR14	PPWD1	DDX42	LSM1	SKIV2L2
PRPF40A	SUGP1	CD2BP2	EIF4A3	PLRG1	PRPF4	WBP11	DHX35	PRPF18	DDX50	LUC7L	TCERG1
PUF60	THRAP3	CDC40	GPATCH1	PPIE	PRPF6	WBP4	DHX8	SLU7	DEK	MATR3	TOE1
RBM10	U2AF1	CDC5L	GPKOW	PPIH	PRPF8	XAB2	FAM32A	SRRM2	DHX38	MOV10	TRIM24
RBM17	U2AF2	CRNKL1	IK	PPIL1	RBM22	ZMAT2	FAM50A	SYF2	DHX9	NCOR1	TTC14
RBM25		CTNNBL1	ISY1	PPIL2	RBMX2		FRA10AC1	WDR83	ERH	NKAP	UBL5
RBM5		CWC15	LSM2	PPIL4	SART1		HNRNPC		FAM58A	PRPF38B	WDR70
SF1		CWC22	LSM4	PQBP1	SNRNP200		LENG1		FUBP3	PRPF39	ZCCHC10

c

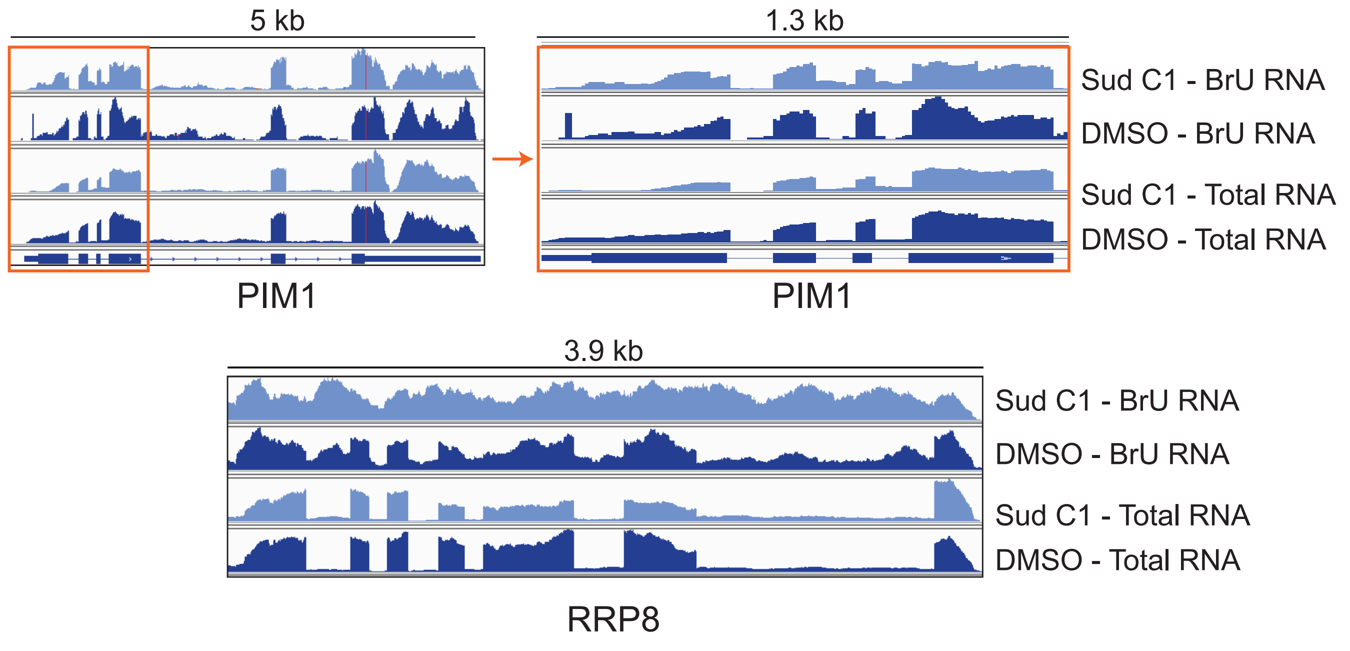

Supplementary Figure 3. Gene ontology analysis of genes displaying splicing changes upon Sud C1 treatment.

a) GOrilla gene ontology analysis was performed with genes affected either by intron retention or exon skipping (with |ΔPIR| and |ΔPSI| ≥ 25, respectively) and a background set of all expressed genes (with at least “vlow” quality label after vast-tool analysis). The top 10 categories of enriched processes are shown, both for total and BrU RNA. b) List of spliceosome components and splicing factors encompassing splicing events with |ΔPSI| or |ΔPIR| ≥ 15 in Sud C1-treated cells (either total RNA or BrU RNA). c) Examples of short transcripts with short affected introns: PIM1 (Pim-1 Proto-Oncogene, Serine/Threonine Kinase) is a known short-lived oncoprotein and RPM8 (Ribosomal RNA Processing 8 Methyltransferase Homolog) contains one of the top differentially retained introns detected in the analysis. A zoomed figure for the first three introns of *PIM1* is also shown. ΔPSI and ΔPSI: delta (treated – control) PSI (Percent Spliced In) and delta PIR (Percent of Intron Retention). While the first displays higher intron retention for very short introns, the second displays more widespread intron retention across the transcript, which is more prominent in BrU-labeled RNA (partly due -as expected- to the isolation of nascent RNA that still has to be fully spliced, as revealed by the higher intronic signal in the DMSO control). These examples illustrate how drug-induced intron retention can occur in transcripts with high biological relevance for cancer cells, as supported also by the gene ontology analyses, and how different ranges of effects can be achieved within a general tendency for retention.

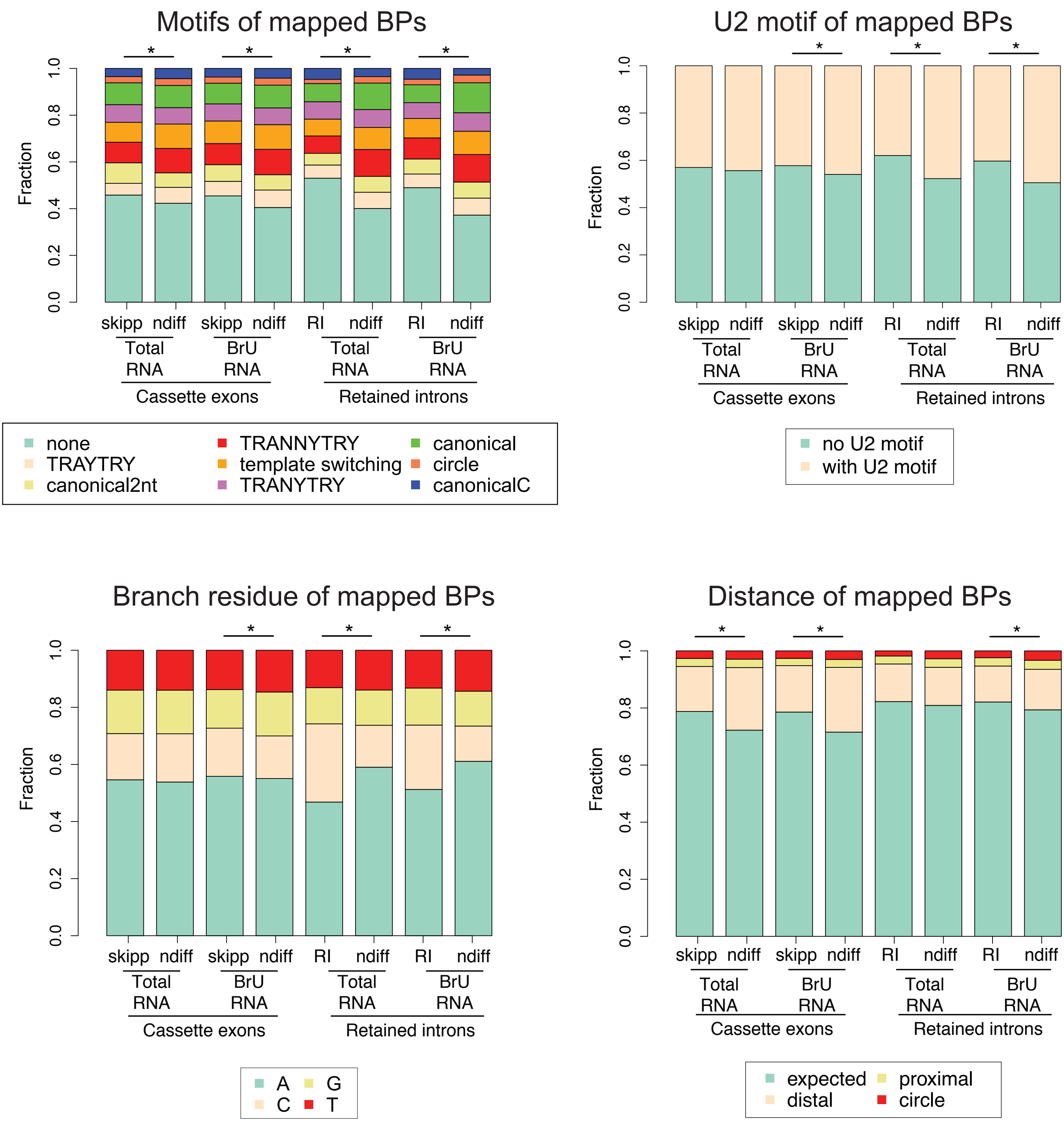

Supplementary Figure 4. Analysis of mapped BPs in affected and non-affected sequences. This analysis was based on features of BPs experimentally verified by Fairbrother's group[5]. Using their genomic coordinates, the experimentally verified BPs were mapped to retained and non-retained introns, or, in the case of cassette exons, to their upstream introns. After mapping, some of the features reported for mapped BPs[5] were compared between the different groups of retained introns / skipped exons vs. non-affected retained introns / cassette exons, as indicated in the figure. P values were determined by permutation tests with 100,000 repeats. *; **; ***: P value < 0.01, 0.001 and 0.0001, respectively.

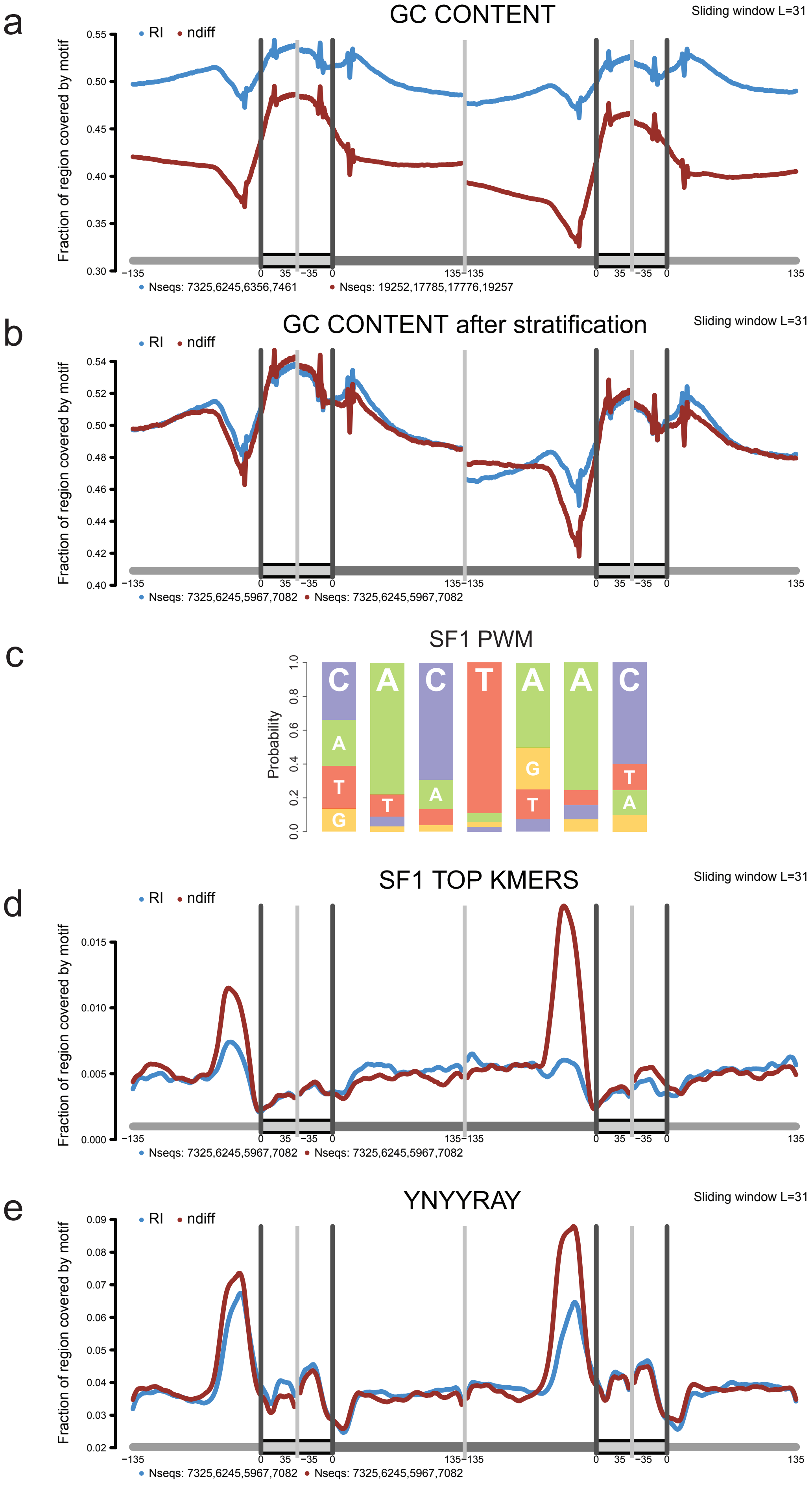

Supplementary Figure 5. RNAmaps for differentially retained introns in BrU RNA.
a) RNAmap for GC content of retained introns (RI, ΔPIR ≥ 25) vs. non-differentially retained (ndiff, -5 ≤ ΔPIR ≤ 5). b) RNAmap for GC content after stratification (i.e. both data sets, RI and ndiff, have been sub-selected to approximately fit GC content). c) Motif logo for SF1 PWM. d) RNAmap for SF1 motif after stratification of the data based on their GC content. The 18 most-likely 7mers according to the SF1 PWM were used and the cumulative probability of these 7mers is approximately 0.25. e) RNAmap for BP motif YNYYRAY after stratification of the data based on their GC content.

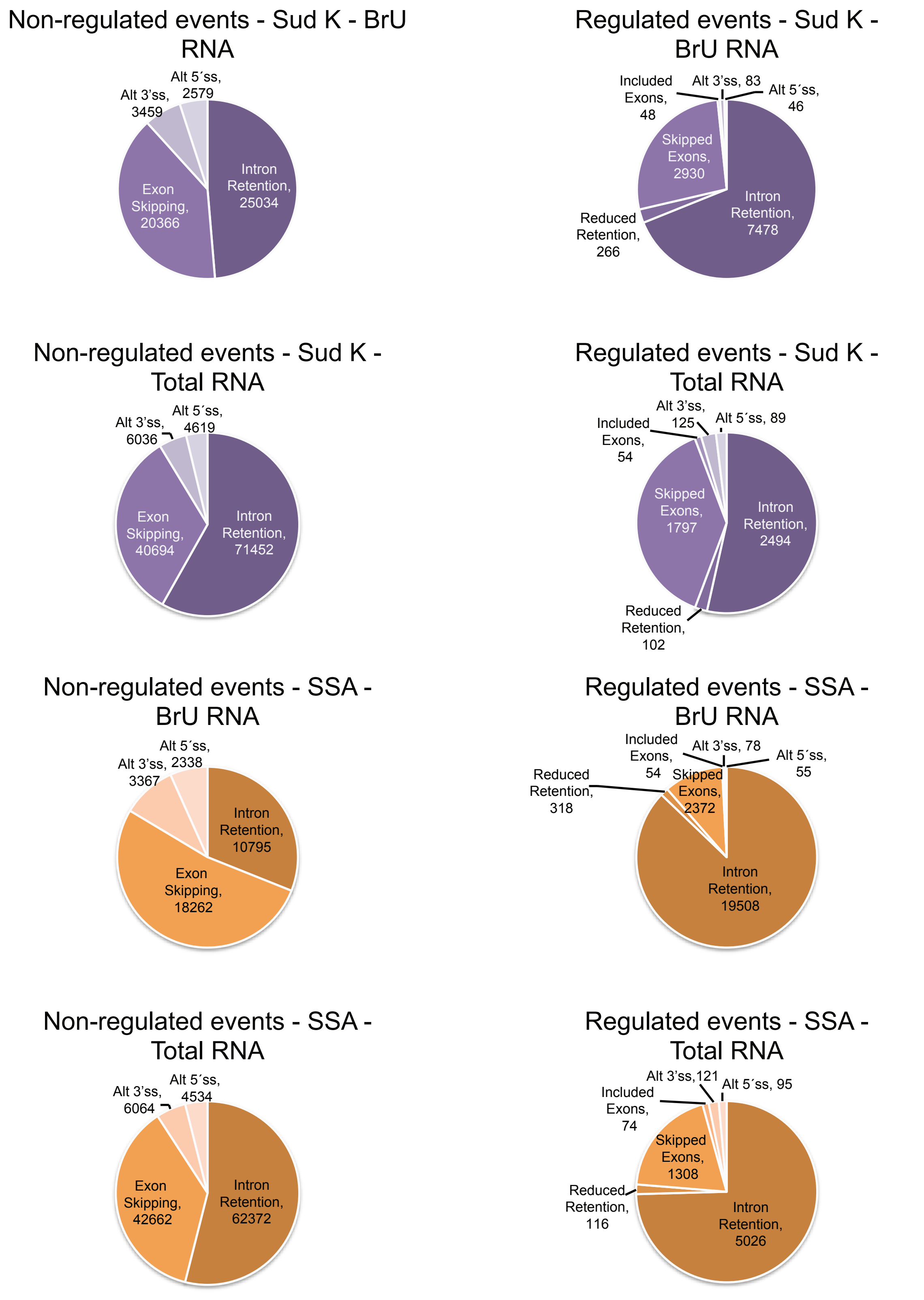

Supplementary Figure 6. Genome-wide analysis of splicing regulation induced by Sud K and SSA.
Number of regulated events with |ΔPSI| ≥ 25 and non-regulated events with |ΔPSI| ≤ 5 detected by analyzing RNA-Seq data of total and BrU RNAs of the indicated conditions.

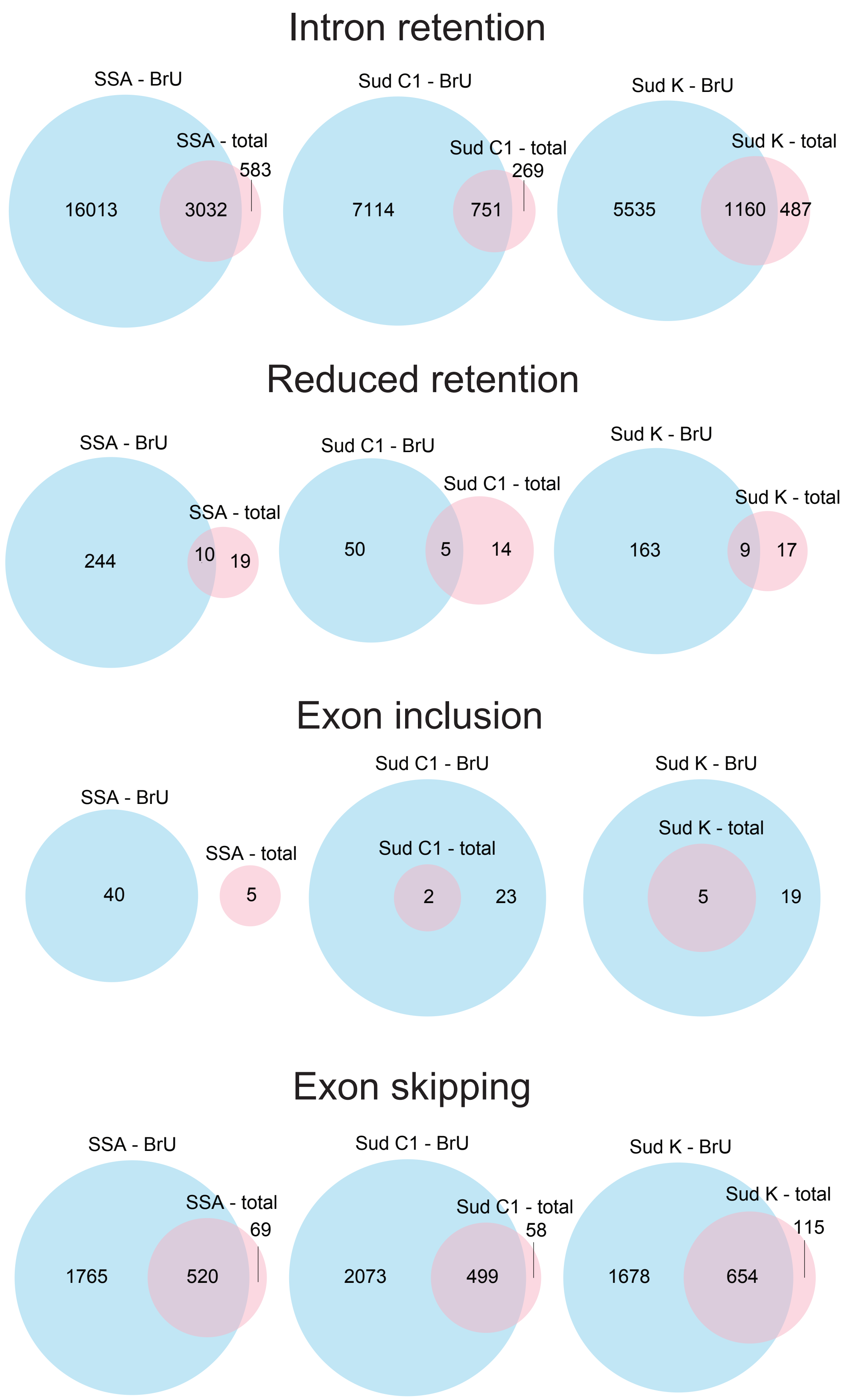

Supplementary Figure 7. Venn diagrams showing the overlaps among effects detected on BrU RNA and total RNA.
All overlaps are significantly larger than expected (χ^2 test P value < 2.2 e-16), except for included exons upon SSA treatment (χ^2 test P value > 0.01).

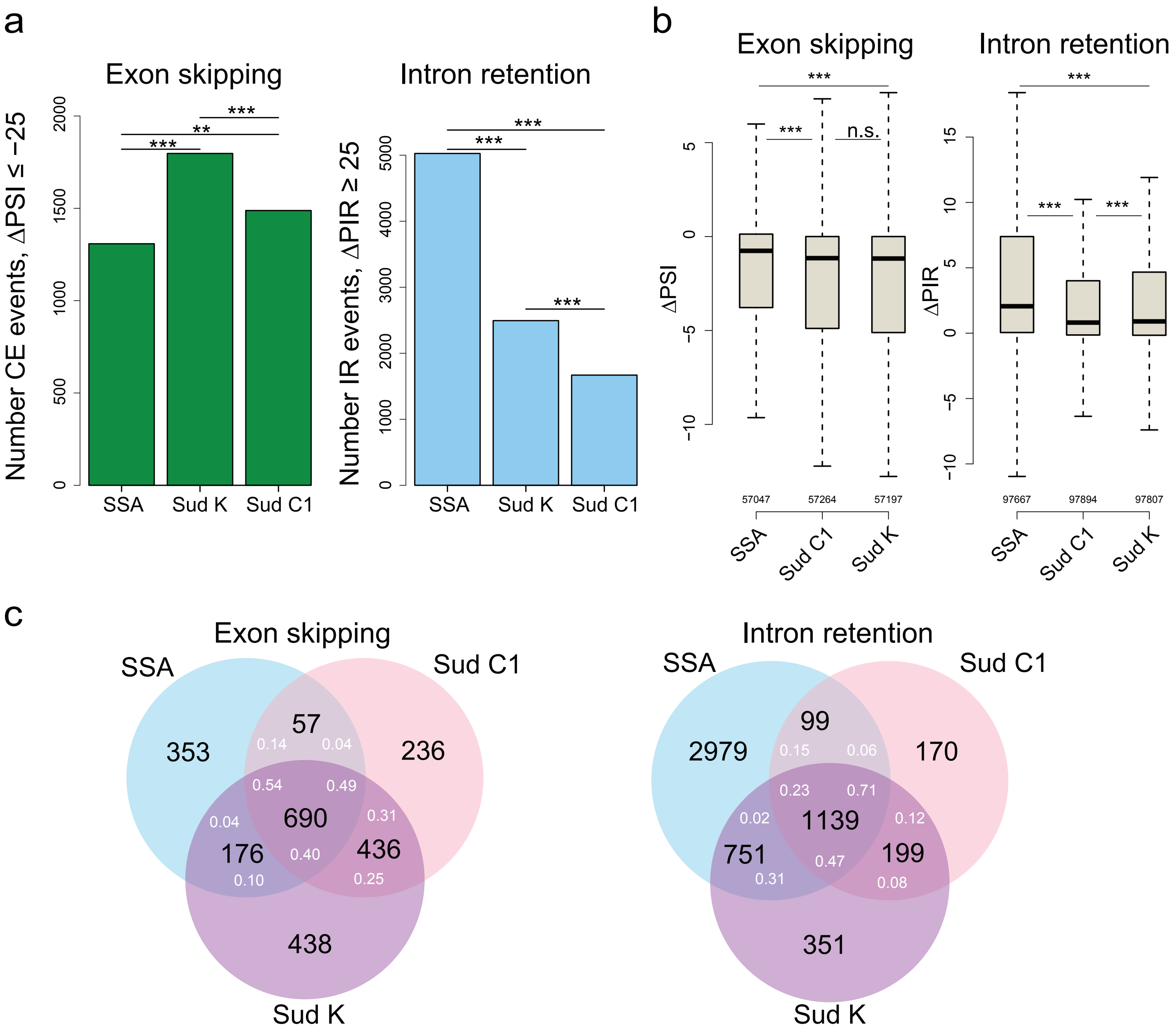

Supplementary Figure 8. Distinct profiles of alternative splicing changes induced by SSA, Sud C1 and Sud K in total RNA.

a) Number of cassette exons and retained introns affected by each of the three drugs in total RNA. *; **; ***: P value < 0.01, 0.001 and 0.0001 from χ^2 tests wrt. all detected cassette exons and retained introns.

b) Distribution of alternative splicing changes for cassette exons and retained introns induced by each of the three drugs in total RNA. *; **; ***: Mann-Whitney U test P value < 0.01, 0.001 and 0.0001, respectively.

c) Overlaps between sets of cassette exons and retained introns affected by each of the three drugs, detected in total RNA data. Numbers in white indicate the ratio of events in common between the closest drug and the drug(s) in the corresponding overlap section.

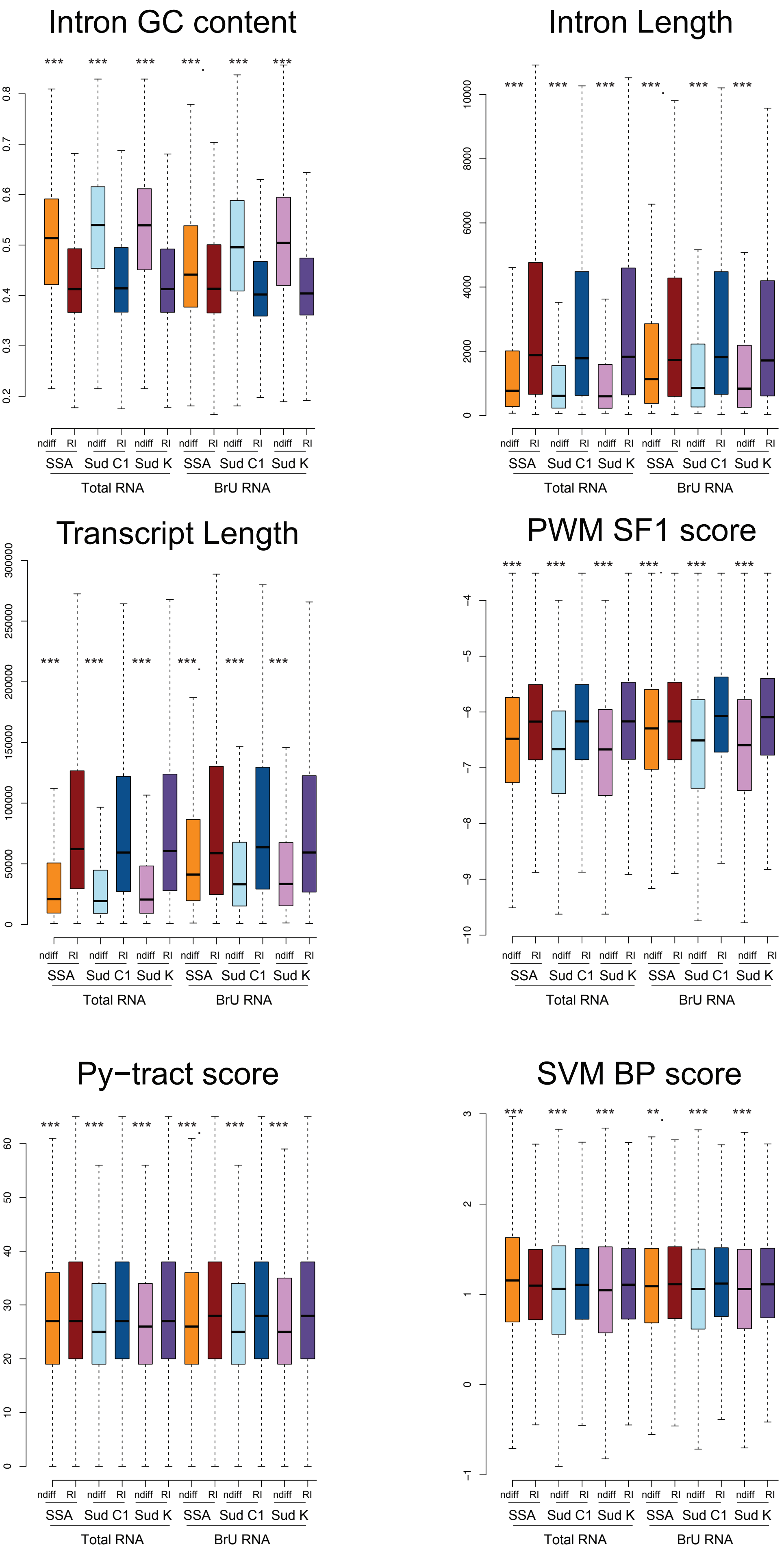

Supplementary Figure 9. Analysis of sequence features linked to intron retention.
Boxplots corresponding to the indicated features extracted with custom scripts (see Methods) are shown, with median values shown by the black line. Outliers were discarded. ndiff: non-differentially spliced introns, with |ΔPIR| ≤ 5; RI: retained introns, with ΔPIR ≥ 25. ΔPIR: delta PIR, i.e. differential Percent of Intron Retention (treated – control). *; **; ***: Mann-Whitney U test P value < 0.01, 0.001 and 0.0001, respectively.

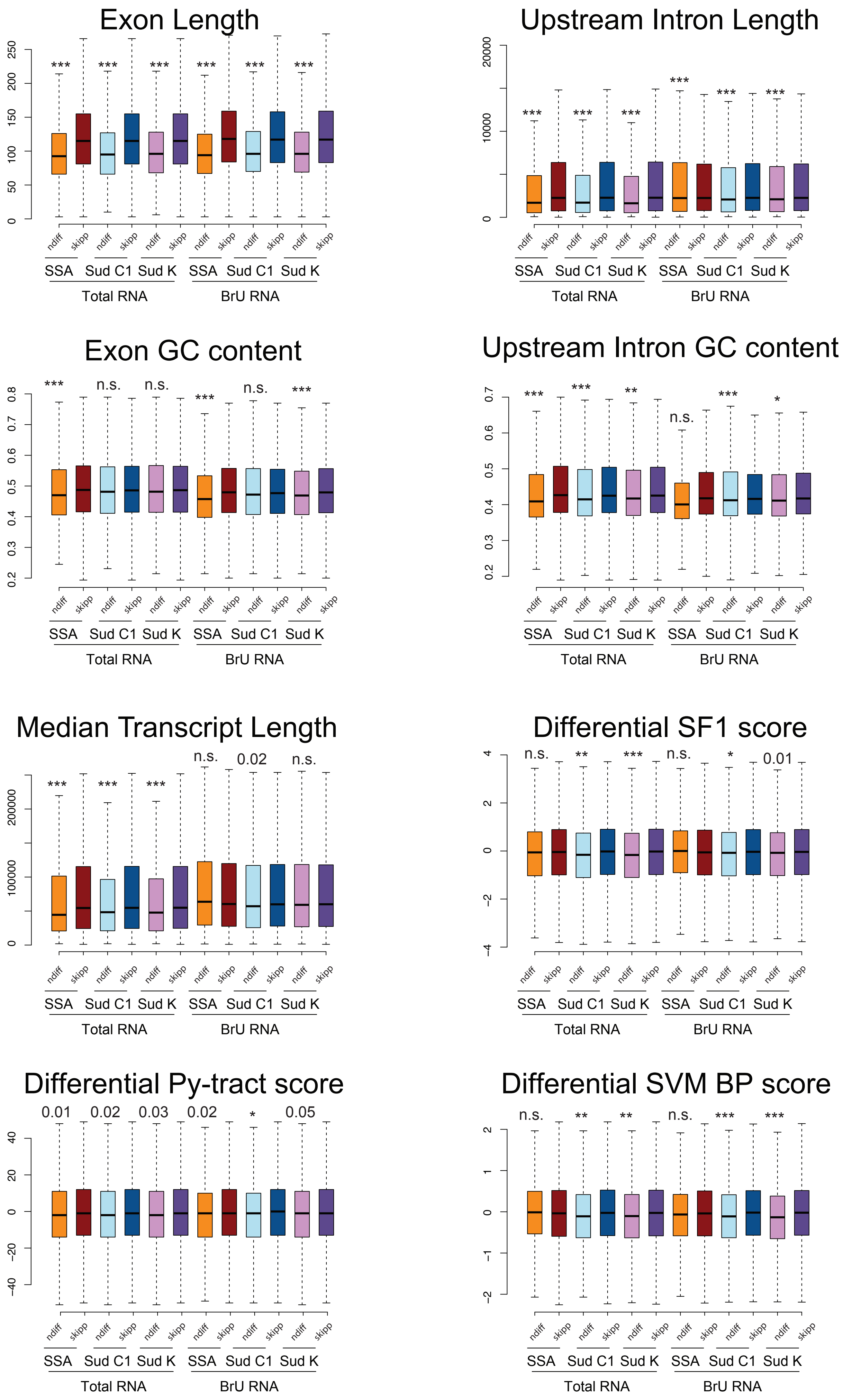
Exon Length
0 50 100 150 200 250
*** *** *** *** *** ***
Upstream Intron Length
0 10000 20000
*** *** *** *** *** ***
Exon GC content
0.2 0.3 0.4 0.5 0.6 0.7 0.8
*** n.s. n.s. *** n.s. ***
Upstream Intron GC content
0.2 0.3 0.4 0.5 0.6 0.7
*** *** ** n.s. *** *
Median Transcript Length
0 100000 200000
*** *** *** n.s. 0.02 n.s.
Differential SF1 score
−4 −2 0 2 4
n.s. ** *** n.s. * 0.01
Differential Py-tract score
−40 −20 0 20 40
0.01 0.02 0.03 0.02 * 0.05
Differential SVM BP score
−2 −1 0 1 2
n.s. ** ** n.s. *** ***
ndiff skipp ndiff skipp ndiff skipp ndiff skipp ndiff skipp ndiff skipp
SSA Sud C1 Sud K SSA Sud C1 Sud K
Total RNA BrU RNA

Supplementary Figures 10. Analysis of sequence features linked to exon skipping.
Boxplots corresponding to the indicated features extracted with custom scripts (see Methods) are shown, with median values shown by the black line. Outliers were discarded.
ndiff: non-differentially spliced cassette exons, with |ΔPSI| ≤ 5; skipp: skipped exons, with ΔPSI ≤ -25. BP features and SF1 binding motif were analyzed in the 3' 150 nucleotides of each intron. For some features (i.e. SF1 score, SVM BP score and Py-tract score), the difference between the values corresponding to the two introns surrounding the exons was calculated and compared among regulated and non-regulated events. ΔPSI: differential PSI (treated – control).
*; **; ***: Mann-Whitney U test P value < 0.01, 0.001 and 0.0001, respectively.

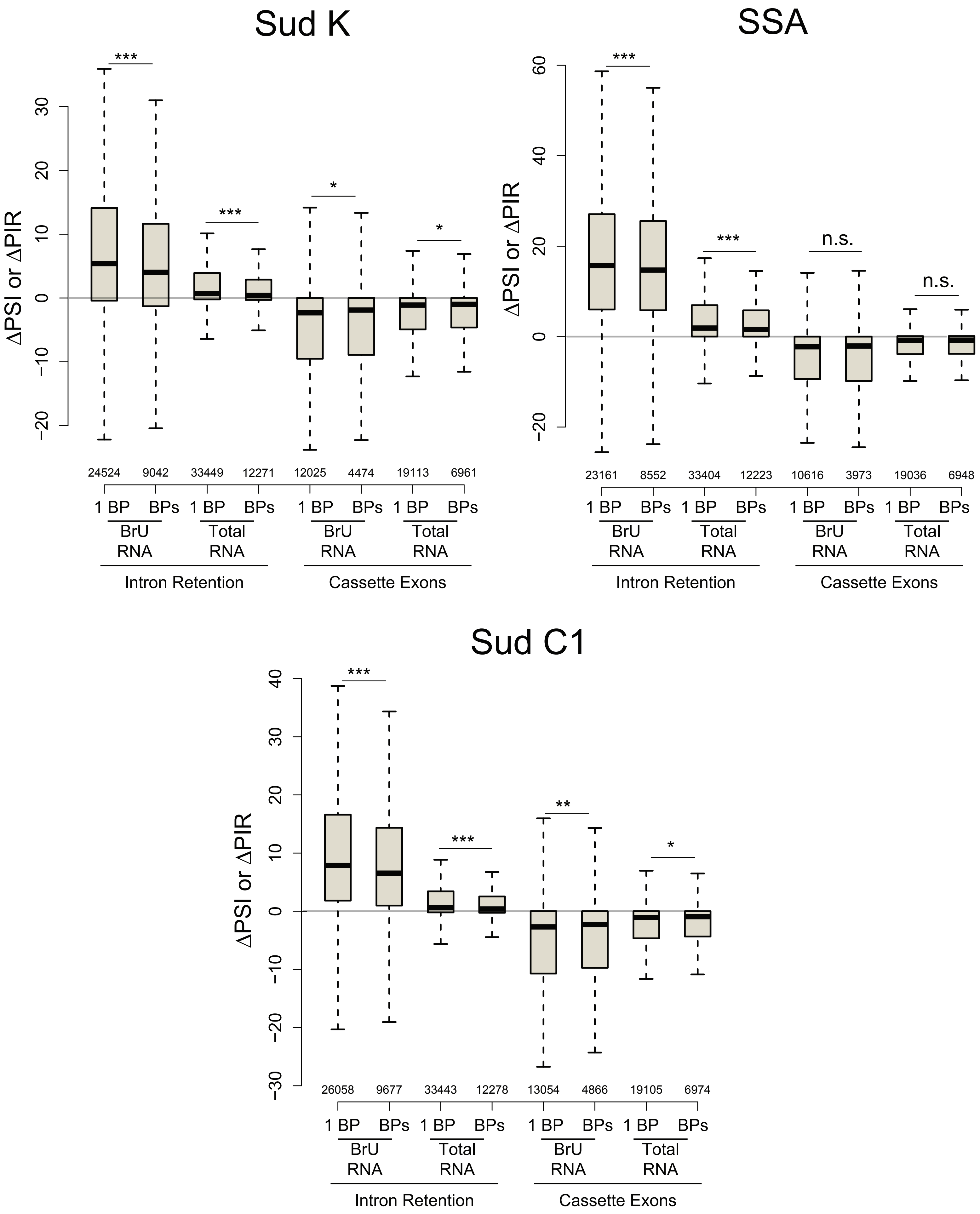

Supplementary Figure 11. Weaker drug effects in the presence of additional BP sequences. Changes in exon inclusion (ΔPSI) or intron retention (ΔPIR) upon treatment with each drug are represented for total and BrU RNA, for 3'ss containing a single (1BP) or multiple (BPs) matches of the BP consensus YNYYRAY within a window of 100 nucleotides from the 3'ss. Numbers of 3'ss considered in each category are also indicated. Differently from Figure 4B, all the plots contain whiskers.
*; **; ***: Mann-Whitney U test P value < 0.01, 0.001 and 0.0001, respectively.

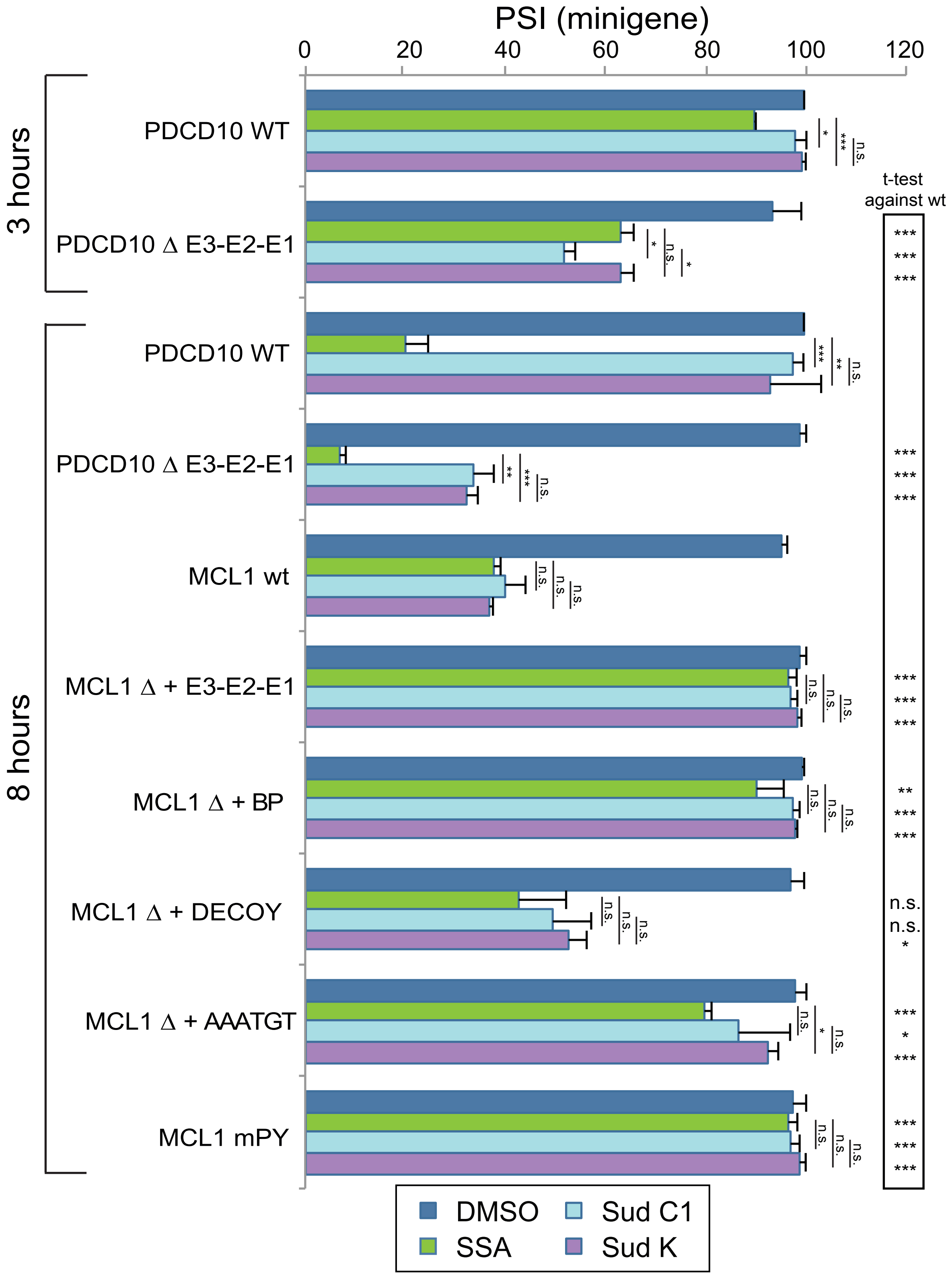

Supplementary Figure 12. Effects of SSA, Sud C1 and Sud K on different minigenes. Cells transfected with the indicated minigenes were treated for 3 or 8 hours, as reported on the left. While the different drugs similarly affect MCL1 and its mutants, SSA has higher effects on PDCD10, especially upon 8 hours treatments. *; **; ***: t-test P value < 0.01, 0.001 and 0.0001, respectively. n.s.: not statistically significant (t-test P value > 0.01). A minimum of three treatment replicates was used for each condition. Error bars represent standard deviation from the mean value.

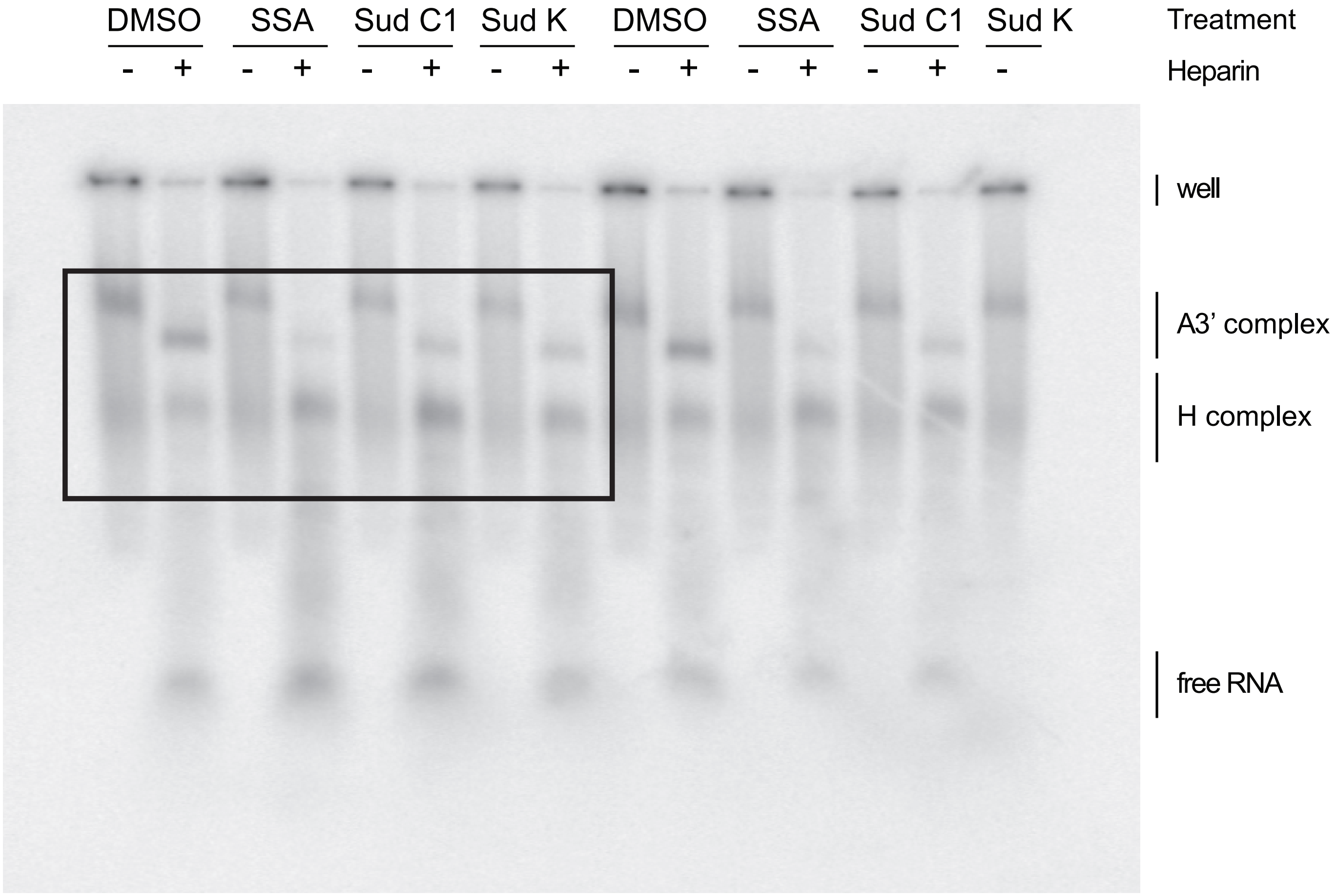

Supplementary Figure 13. Uncropped image of A3´complex assembly with different drugs shown in Figure 5c.
The black rectangle highlights the area of the image shown in Figure 5c.

Supplementary References

1 Gao, K., Masuda, A., Matsuura, T. & Ohno, K. Human branch point consensus sequence is yUnAy. *Nucleic Acids Res* **36**, 2257-2267 (2008).

2 Mercer, T. R. *et al.* Genome-wide discovery of human splicing branchpoints. *Genome Res* **25**, 290-303 (2015).

3 Corvelo, A., Hallegger, M., Smith, C. W. & Eyras, E. Genome-wide association between branch point properties and alternative splicing. *PLoS Comput Biol* **6**, e1001016 (2010).

4 Jeck, W. R. *et al.* Circular RNAs are abundant, conserved, and associated with ALU repeats. *RNA* **19**, 141-157 (2013).

5 Taggart, A. J. *et al.* Large-scale analysis of branchpoint usage across species and cell lines. *Genome Res*. 27, 639-649 (2017).

Supplementary Tables

Table 1. Summary of minigenes used in the study
Table 2. List of oligonucleotide primers used in the study

Supplementary Data

Supplementary Data 1. Splicing analysis of RNA-Seq data showing PSI (or PIR) values and quality scores for all treatment conditions. Results are in hg19.

Supplementary Data 2. Gene expression analysis of RNA-Seq data, showing RPKM values across all treatment conditions.

		DMSO		SudC1			VIGEVANI ET AL SUPPLEMENTARY TABLE 1 - Minigenes and results
#	Minigene	Average Inclusion _DMSO (%)	StDev_ DMSO	Average Inclusion _SudC1 (%)	StDev_ SudC1	First intron lentgh	First intron sequence (last 100nt)
1	PDCD10 WT	98,60	2,26	96,46	2,28	546	attaagagtgtacttaatttctttcaaatgtagctgatgttcttttctctcacaaatttctttcaaatgtagctgatattctttattcttttcccaacag
2	PDCD10 Δ E1	96,50	3,11	71,63	3,66	524	attaagagtgtactgtgattagattaagagtgtacttaatttctttcaaatgtagctgatgttcttttctagctgatattctttattcttttcccaacag
3	PDCD10 Δ E1-E2	95,62	5,35	58,23	5,92	509	attgcgcttaacataattaagagtgtactgtgattagattaagagtgtacttaatttctttcaaatgtagagctgatattctttattcttttcccaacag
4	PDCD10 Δ E1-E2-E3	84,44	14,62	31,52	17,28	486	aaaggctggctgttcttgaagtgattgcgcttaacataattaagagtgtactgtgattagattaagagtgagctgatattctttattcttttcccaacag
5	MCL1 wt	90,91	6,00	7,06	6,64	351	TAAATCCCAGTAGCGATTTTCCCGCCGCGGGTGGGCAGGCGAATCTTGCGCCGGTTTAGACAAAGGAGGCCGTGAGGACCTGCATGCTTTTCTTTCTCAG
6	MCL1 Δ	97,76	0,69	10,59	12,19	275	TTTGGAAATGGCAGCTCTTGTTCAAAGACCGGAAAGGGTGGGATGTCAATTTCAAGTGGGGTCAACCTGAGTTGAGGACCTGCATGCTTTTCTTTCTCAG
7	MCL1 Δ + upstream PDCD10	97,41	1,33	21,26	5,64	356	tccccactccaaccagctagccgaaaaggctggctgttcttgaagtgattgcgcttaacataattaagagtgtGAGGACCTGCATGCTTTTCTTTCTCAG
8	MCL1 Δ + E3-E2-E1	96,58	0,72	85,28	10,01	356	tagattaagagtgtacttaatttctttcaaatgtagctgatgttcttttctctcacaaatttctttcaaatgtGAGGACCTGCATGCTTTTCTTTCTCAG
9	MCL1 Δ + BP	100,00	0,00	69,66	7,60	282	ATGGCAGCTCTTGTTCAAAGACCGGAAAGGGTGGGATGTCAATTTCAAGTGGGGTCAACCTGAGTTctctcacGAGGACCTGCATGCTTTTCTTTCTCAG
10	MCL1 Δ + DECOY	83,36	9,18	4,97	0,51	281	AATGGCAGCTCTTGTTCAAAGACCGGAAAGGGTGGGATGTCAATTTCAAGTGGGGTCAACCTGAGTTctctccGAGGACCTGCATGCTTTTCTTTCTCAG
11	MCL1 Δ + Y-rich + AAATGT	97,96	2,09	66,89	15,49	289	CTCTTGTTCAAAGACCGGAAAGGGTGGGATGTCAATTTCAAGTGGGGTCAACCTGAGTTtttctttcaaatgtGAGGACCTGCATGCTTTTCTTTCTCAG
12	MCL1 Δ + Y-rich + AAATGT - BP	100,00	0,00	99,65	0,60	296	TCAAAGACCGGAAAGGGTGGGATGTCAATTTCAAGTGGGGTCAACCTGAGTTtttctttcaaatgtctctcacGAGGACCTGCATGCTTTTCTTTCTCAG
13	MCL1 Δ + Y-rich	99,16	0,88	16,71	5,01	284	GGCAGCTCTTGTTCAAAGACCGGAAAGGGTGGGATGTCAATTTCAAGTGGGGTCAACCTGAGTTtttctttctGAGGACCTGCATGCTTTTCTTTCTCAG
14	MCL1 Δ + AAATGT	99,68	0,08	65,31	4,91	281	AATGGCAGCTCTTGTTCAAAGACCGGAAAGGGTGGGATGTCAATTTCAAGTGGGGTCAACCTGAGTTaaatgtGAGGACCTGCATGCTTTTCTTTCTCAG
15	mMCL1 wt	88,59	2,17	85,24	18,57	273	TAGAGCCGGGAGAGCACGGTCCCCTCGTCGTGGGTGGGCAGAAGGGTAGTGCCCCGCTGCAGACAAAGGAGGCCATGAGGTTTCTTGCTTTTCTTCTCAG
16	m-hMCL1 chimera 1	97,63	3,36	99,30	0,99	349	AAATCCCAGTAGCGATTTTCCCGCCGCGGGTGGGCAGGCGAATCTTGCGCCGGTTTAGACAAAGGAGGCCGTCCATGAGGTTTCTTGCTTTTCTTCTCAG
17	m-hMCL1 chimera 2	95,46	0,62	10,99	1,54	348	TAAATCCCAGTAGCGATTTTCCCGCCGCGGGTGGGCAGGCGAATCTTGCGCCGGTTTAGACAAAGGAGGCCGTGAGGACCTGCATGCTTTTCTTTCTCAG
18	hMCL1 G-29A	97,91	0,34	44,96	5,59	351	TAAATCCCAGTAGCGATTTTCCCGCCGCGGGTGGGCAGGCGAATCTTGCGCCGGTTTAGACAAAGGAGGCC**A**TGAGGACCTGCATGCTTTTCTTTCTCAG
19	mMCL1 A-26G	100,00	0,00	97,48	4,37	273	TAGAGCCGGGAGAGCACGGTCCCCTCGTCGTGGGTGGGCAGAAGGGTAGTGCCCCGCTGCAGACAAAGGAGGCC**G**TGAGGTTTCTTGCTTTTCTTCTCAG
20	hMCL1 mPY	97,26	2,23	92,72	1,14	355	TCCCAGTAGCGATTTTCCCGCCGCGGGTGGGCAGGCGAATCTTGCGCCGGTTTAGACAAAGGAGGCCGTGAGGACCTGCATTTCTTGCTTTTCTTCTCAG
21	hMCL1 mPY Δ ACCTGCA	84,78	2,46	80,58	3,53	348	CTGTAAATCCCAGTAGCGATTTTCCCGCCGCGGGTGGGCAGGCGAATCTTGCGCCGGTTTAGACAAAGGAGGCCGTGAGGTTTCTTGCTTTTCTTCTCAG
22	MCL1 A-26C	63,94	6,94	4,04	0,52	351	TAAATCCCAGTAGCGATTTTCCCGCCGCGGGTGGGCAGGCGAATCTTGCGCCGGTTTAGACAAAGGAGGccgtg**c**gGACCTGCATGCTTTTCTTTCTCAG
23	MCL1 -31/-25 TACTAAC	99,41	1,03	100,00	0,00	351	TAAATCCCAGTAGCGATTTTCCCGCCGCGGGTGGGCAGGCGAATCTTGCGCCGGTTTAGACAAAGGAGGtactaacGACCTGCATGCTTTTCTTTCTCAG
24	MCL1 Δ ACCTGCA	11,85	2,42	4,35	0,40	344	AGTTCTGTAAATCCCAGTAGCGATTTTCCCGCCGCGGGTGGGCAGGCGAATCTTGCGCCGGTTTAGACAAAGGAGGCCGTGAGGTGCTTTTCTTTCTCAG

Vigevani et al — Supplementary Table 2 — List of oligonucleotide primers used in this study

Target	Aim of the Primer Pair	Alternative region	Forward primer	Reverse primer	Products size (bp)
ARRDC3	Exon skipping assay	exon 3	AATTCCGAAGAAGGCTTCCA	GTATAGCCCTTCCTTTCAAT	320 - 172
MCL1	Exon skipping assay	exon 2	AGACCTTACGACGGGTTGG	ACCAGCTCCTACTCCAGCAA	401 - 153
RBM5	Exon skipping assay	exon 16	CGGCTGTAGTGTCCCAGAGT	TTGCGAGTTGGGGTCATAAT	239 - 154
GADD45	Intron retention assay	intron 1	AATATGACTTTGGAGGAATTC	AGTGATCGTGCGCTGACTC	600 - 114
DRP2	Intron retention assay	intron 12	AACTCCGCAGAGTCCAGAAA	GCCTCTTTCCTCCTCCAAAC	1091 - 167
ZBTB17	Intron retention assay	intron 14	GGTCACTGTGGATGACATGG	CTTCCTGCACTTGCTTCACA	266 - 179 - 158
PHF5A	Exon skipping assay	exon 2	gtggccggcttagttaggag	ggacgcacataggagtcaca	138 - 114
mMCL1	Exon skipping assay	exon 2	GACGACCTATACCGCCAGTC	AAAGCCAGCAGCACATTTCT	491 - 243
Minigenes (PT1-PT2)	Minigene AS		GTCGACGACACTTGCTCAAC	AAGCTTGCATCGAATCAGTAG	
MCL1 - endogenous	Endogenous AS in minigenes assay	exon 2	ATCTGGTAATAACACCAGTACGGAC	ACCAGCTCCTACTCCAGCAA	586 - 338
mMCL1 - minigene	Minigene AS	exon 2	GTCGACGACACTTGCTCAAC	AAAGCCAGCAGCACATTTCT	641 - 393
PDCD10 - minigene	Minigene AS	exon 7	GTCGACGACACTTGCTCAAC	AAGCTTGCATCGAATCAGTAG	350 - 271
PDCD10 - endogenous	Endogenous AS in minigenes assay	exon 7	CTTCGTATGGCAGCTGATGA	CAGAGTATCACTGAAACTTTTGG	294 - 215
AdML 3'	T7 template for *in vitro* transcription	intron 1	GCTAATACGACTCACTATAGGGTGATGATGTCATACTTATC	CCCACTGGAAAGACCGCGAAGA	81
MCL1 - external primers	BP mapping	intron 1	TTTTGGAAATGGCAGCTCTT	GTGAGTCCGGGGAGAGATG	<239
MCL1 - nested primers	BP mapping	intron 1	AGGGTGGGATGTCAATTTCA	AAAAAGGGAGTGAGGCCTTG	<183
PDCD10 - external primers	BP mapping	intron 6	TAAAATCCCCACTCCAACCA	TCTGAAACCAAACGCCATAA	<355
PDCD10 - nested primers	BP mapping	intron 6	TGCGCTTAACATAATTAAGAGTG	TCGGAAGTACTTTTAAGAAAAGAAGAA	<183
