# Supplementary material for: Molecular basis of differential 3′ splice site sensitivity to anti-tumor drugs targeting U2 snRNP
Source: Nat Commun. 2017 Dec 13;8:2100. doi: 10.1038/s41467-017-02007-z (PMC5727392; doi:10.1038/s41467-017-02007-z)
Supplement: Supplementary file 3 — Description of Additional Supplementary Files [file 41467_2017_2007_MOESM3_ESM.pdf]

Descriptions of Additional Supplementary Files

File Name: Supplementary Dataset 1
Description: Splicing analysis of RNA-Seq data showing PSI (or PIR) values and quality scores for all treatment conditions. Results are in hg19.

File Name: Supplementary Dataset 2
Description: Gene expression analysis of RNA-seq data, showing RPKM values across all treatment conditions.
